# Treatment choices: insights into disease-specific use and self-perceived benefits of traditional, complementary and integrative medicine in Germany

**DOI:** 10.1186/s12906-026-05496-y

**Published:** 2026-09-29

**Authors:** Tido von Schoen-Angerer, Manfred Wischnewsky, Michael Jeitler, Benno Brinkhaus, Miriam Ortiz, Rasmus Hoffmann, Christian S. Kessler

**Affiliations:** 1 Charité Competence Center for Traditional and Integrative Medicine (CCCTIM), Charité – University Medical Center Berlin, corporate member of Freie Universität Berlin and Humboldt-Universität zu Berlin, Berlin, Germany; 2 Multidisciplinary Center for Integrative Medicine, Geneva University Hospitals, Geneva, Switzerland; 3 Faculty of Mathematics and Computer Science, University of Bremen, Bremen, Germany; 4 Institute of Social Medicine, Epidemiology and Health Economics, Charité – University Medical Center Berlin, corporate member of Freie Universität Berlin and Humboldt-Universität zu Berlin, Berlin, Germany; 5 Institute for General Practice and Interprofessional Care, University Hospital Tuebingen, Tuebingen, Germany; 6 Robert Bosch Center for Integrative Medicine and Health, Bosch Health Campus, Stuttgart, Germany; 7 Institute for Sociology, Otto-Friedrich-University Bamberg, Bamberg, Germany; 8 Department of Internal Medicine and Nature-Based Therapies, Immanuel Hospital Berlin, Berlin, Germany; 9 Department of Integrative Health Care and Prevention, University of Augsburg & University Hospital Augsburg, Augsburg, Germany

**Keywords:** Health care utilization, Delivery of health care, Traditional medicine, Complementary medicine, Integrative medicine, Online-representative, Cross-sectional study

## Abstract

**Background:**

Previous cross-sectional studies suggest that Traditional, Complementary and Integrative Medicine (TCIM) is used frequently in Germany. We assessed TCIM usage patterns across different disease categories in the German population.

**Method:**

In this cross-sectional survey of an online-representative sample of the German population aged 18–75 years, participants were asked about their use and acceptance of TCIM. Participants were also queried about 15 illness categories. Health-related quality of life was assessed using the EQ-5D-5L questionnaire. Data were analyzed descriptively and inferentially.

**Results:**

A total of 4,065 individuals (52% female) responded. 70% had used TCIM at some point in their lives, 32% in the last 12 months, and 18% were current users. Over half (52%) reported a mostly or very positive attitude towards TCIM. The highest rates of TCIM use were reported for acute respiratory illness (55.9%), chronic gastrointestinal illness (51.4%) and acute gastrointestinal illness (49.2%). The illness-specific, self-reported benefit from TCIM ranged from 61.4 to 86.2% (mean 70.8%) and remained high even among individuals of poorer health-related quality of life. No relation between TCIM use across different disease categories and the global research activity in those categories was apparent in the scatter plot.

**Conclusions:**

TCIM is widely used and perceived as beneficial for acute and chronic conditions in Germany. Individuals with poorer health status also reported substantial benefit. Stakeholders in the German healthcare system should critically examine the high level of TCIM use, promote rigorous scientific evaluation, integrate evidence-informed TCIM with conventional medicine, and ensure appropriate reimbursement for TCIM through statutory health insurance.

**Supplementary Information:**

The online version contains supplementary material available at https://doi.org/10.1186/s12906-026-05496-y.

## Background

Health systems need to be responsive to the expectations of the populations they serve [1]. As Traditional, Complementary and Integrative Medicine (TCIM) is extensively used globally [2], health systems must address this demand appropriately. The WHO Global Traditional Medicine Strategy 2025–2034 sets the objective to “integrate safe and effective TCIM into health systems” [3]. Detailed, concept-specific knowledge of people's current use patterns and views of TCIM is therefore essential.

In Europe, TCIM use is widespread (27.7% use in the last 12 months) with wide variations between countries [4], and there are many TCIM research and teaching institutions [5]. Several TCIM systems originated in Europe, including Ancient Greek humoral theory, Hildegard von Bingen’s medieval medicine, 18th century homeopathy, 19th century natural healing methods, such as Priessnitz’ and Kneipp’s hydrotherapy with cold water, and 20th century anthroposophic medicine. Paradoxically, Europe ranks lowest worldwide in the establishment of national policies for TCIM: only 36% of countries in the WHO Europe region had a national TCIM policy in place, compared with 44–100% in other regions of the world [2, 6]. This discrepancy underscores the need for European countries to align with WHO objectives and to adequately evaluate TCIM and integrate it into health systems.

The aim of this online-based cross-sectional study was to evaluate use patterns and self-perceived benefits of TCIM in Germany across different disease categories. The rationale was that granular disease-specific data are necessary to inform the better integration of TCIM into the German health care systems, and potentially other European countries.

## Methods

A comprehensive questionnaire was developed under the coordination of the Charité University Outpatient Clinic for Complementary and Integrative Medicine at the Immanuel Hospital Berlin and the Institute of Social Medicine, Epidemiology, and Health Economics of Charité—University Medical Center Berlin (see supplementary material) [7]. 

The overall survey aimed to update previous findings on the use and acceptance of TCIM in Germany and to explore additional dimensions. The full questionnaire covered sociodemographics, TCIM use, attitudes toward TCIM, diagnoses for which TCIM was used, familiarity with relevant terminology, attitudes and behavior toward TCIM, the role of TCIM during the Covid-19 pandemic, nutrition, Ayurveda, Sinus milieu indicator and health-related quality of life. Not all survey data are reported in this paper.

The questionnaire used the following TCIM terms: “Naturheilkunde” (literal translation of the German: knowledge of natural healing); complementary medicine; alternative medicine; and integrative medicine, each accompanied with brief definitions. For questions referring to the entire field of TCIM, the cumulative formulation “Naturheilkunde/natural therapies/complementary medicine/integrative medicine or alternative medicine” was used.

**Fig. 1:**
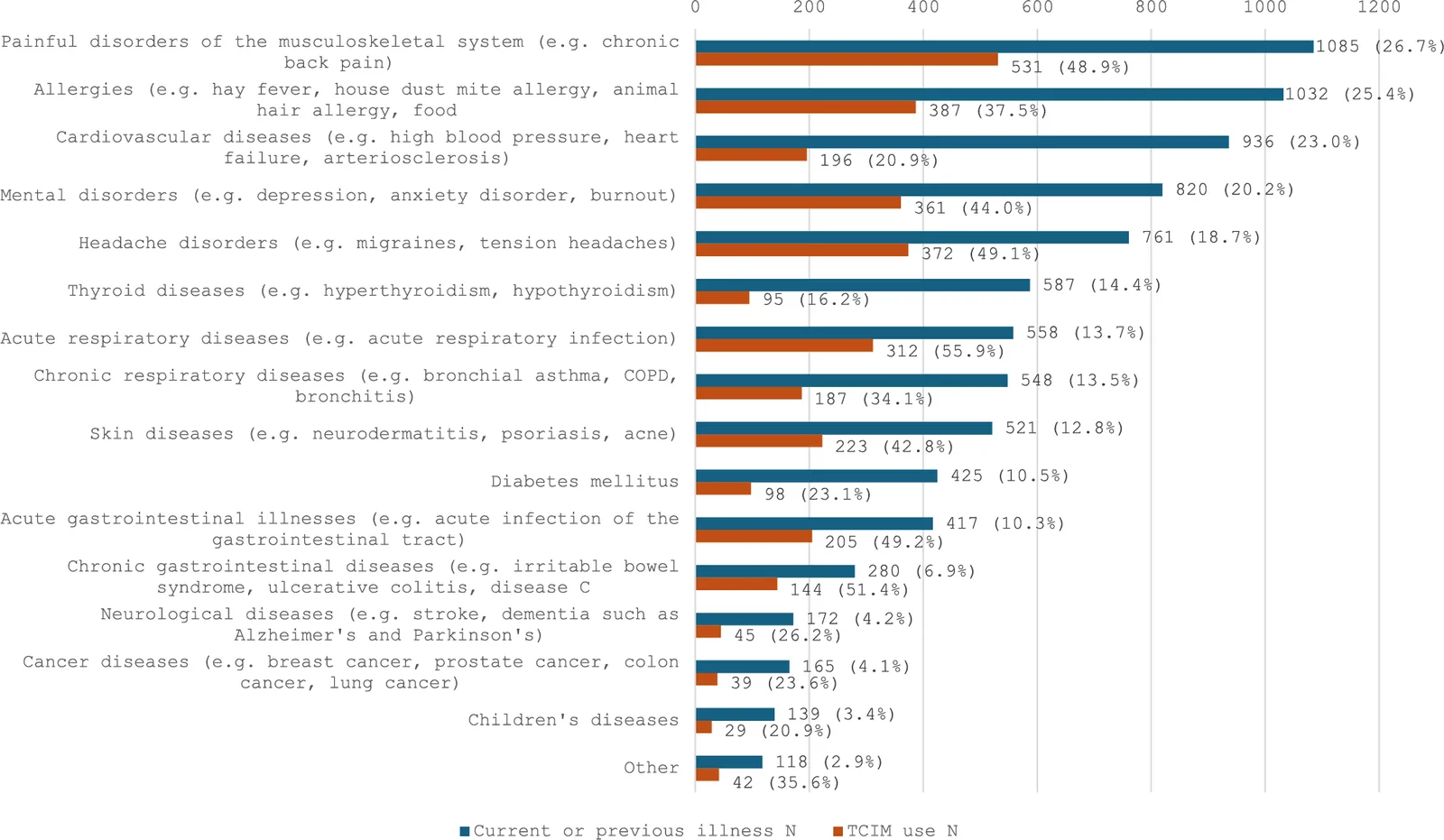
Reported current or past illnesses and TCIM use for specific illnesses. Percentages: reported illnesses, of all respondents (*n* = 4,065); TCIM use, of respondents reporting each illness. Multiple illnesses could be reported

The questionnaire included 15 illness categories; for each category several conditions were listed as examples (see Fig. 1).

General health status was assessed as health-related quality of life (HRQoL) with the EQ-5D-5L questionnaire. The questionnaire includes 5 questions to assess the current ability for the dimensions mobility, self-care, usual activities, pain and discomfort, anxiety and depression by rating them on a scale of “no problems, slight, moderate, severe problems and extreme problems". It also includes a visual analogue scale (0-100) for self-rated health and a general health rating (excellent, very good, good, fair, poor).

The survey was conducted as a cross-sectional, online-survey between September and October 2022 among the German-speaking residential population aged 18–75 years. Inclusion criteria included active consent to the informed consent form, age *≥* 18 years, sufficient German proficiency, and cognitive ability to complete an online survey.

The survey was administered by the German market research institute Respondi under supervision of the study team. The respondents were recruited from Respondi’s online access panel. The survey was conducted online using Computer Assisted Web Interview (CAWI). The study design has been described in further detail elsewhere [7]. 

Three illnesses were identified for subgroup-analysis: acute respiratory illness, allergy, and musculoskeletal pain. These represented the most frequently reported illnesses within each of three predefined illness groups: (i) acute illnesses (acute respiratory illnesses, acute gastro-intestinal illness and pediatric illnesses); (ii) chronic illnesses with likely intermittent symptoms (allergies, headaches, diabetes, thyroid illnesses); and (iii) chronic illnesses with likely ongoing symptoms (musculoskeletal pain, mental illness, cardiovascular illness, chronic respiratory illness and chronic gastrointestinal illness).

Analyses among individuals with these three illnesses focused on those with poor or fair HRQoL (sample-specific thresholds provided in the result section).

To assess whether TCIM usage frequency for a given illness corresponded to the volume of TCIM research activity (as expressed in number of systematic reviews), we used the evidence map by Ang et al. [8]. Survey illness categories were converted into ICD-11 disease categories for comparability to Ang et al.’s evidence map. If a survey category matched more than one ICD-11 category, the most common illness in that survey category determined the ICD-11 category (e.g. hay fever is the most common form of allergy; as hay fever belongs to the ICD-11 category diseases of the respiratory system, the survey category allergy was classified as ICD-11 disease of the respiratory system – see conversion table as supplementary table).

### Ethics approval and consent to participate

The study was approved by the Charité Ethics Committee (EA2/128/22) and registered with ClinicalTrials.gov (NCT05530720). Informed consent was obtained from all participants. The collection, processing and storage of all data generated in the study were carried out in accordance with the international guidelines for clinical trials (Declaration of Helsinki, ICH-GCP) and the research ethics framework of the accompanying sociological research.

### Statistical analysis

The EQ-5D-5L questionnaire responses on dimensions were converted to an index value for which we used the German Ludwig value set version 2.1, updated April 8, 2021.

Normality of distribution was assessed by the Shapiro–Wilk test. Continuous variables were tested for differences with the Mann–Whitney U test or the Wilcoxon signed-rank test, and categorical variables with the Pearson’s χ2 or the Fisher’s exact test. Continuous variables were given as mean ± standard deviation (SD), or median and interquartile range, and categorical variables as frequencies (percentages). Crosstab analysis revealed patterns, correlations and trends among categorical (nominal or ordinal) variables. Decision trees (DTs) were used as a non-parametric supervised learning method for classification and regression for the in-depth analyses of the three illnesses mentioned above. The aim was to create a model that predicts the value of a target variable by learning simple decision rules inferred from the data features. A scatter plot was used to graphically display correlations between TCIM usage and research activity.

In this study, we do not report demographic weighting (age, gender, education, federal state, and city size). The bias would only be minimally reduced, and we found no statistically significant differences between weighted and unweighted results.

All statistical analyses were performed using R (R Foundation, version 4.3) and SPSS (IBM^®^ SPSS^®^ Statistics, version 29). Further details on statistical methods have been previously described [7, 9]. 

## Results

A total of 4,065 participants responded to the survey (representing a response rate of 21.5%), 51.7% identified as female, 47.9% as male, 0.4% as diverse. Average age was 49.3 ± 15.8 years. Detailed demographics have been reported elsewhere [7]. 70% responded that they had used TCIM at some point in their lives, 32% in the last 12 months and 18% currently. 52% had a mainly positive or very positive attitude towards TCIM; 63% had a mainly positive or very positive attitude towards conventional biomedicine [7]. 

A total of 3,067 respondents reported at least one current or past illness (multiple responses permitted). The three most common illnesses were musculoskeletal pain, allergies, and cardiovascular diseases, reported by 26.7%, 25.4% and 23.0% of respondents respectively (Fig. 1).

The rate of TCIM use was highest for acute respiratory illnesses (55.9%), chronic gastrointestinal illness (51.4%), acute gastrointestinal illnesses (49.2%); and lowest for thyroid diseases (16.2%), cardiovascular diseases (20.9%) and childhood illnesses (20.9%). The highest absolute number of TCIM users were for musculoskeletal pain, allergies and headaches (Fig. 1).

Crosstabulation of illnesses and TCIM use showed that individuals reporting use of TCIM for a given illness, often used TCIM for other illnesses too (Table 1).

**Table 1:** Crosstabulation of reported illnesses and illness-specific TCIM use

Diseases	For which diseases have you already used TCIM?																	
	Acute respir. diseases	Acute gastroint. diseases	Allergies	Chronic resp. diseases	Chronic gastroint. diseases	Diabetes mellitus	Skin diseases	Cardiovascular diseases	Children s diseases	Headache disorders	Cancer	Neurological diseases	Mental idisorders	Thyroid diseases	Musculoskeletal pain	Others	No TCIM use for listed diseases	Total
Acute resp. diseases	**312**	99	86	63	26	18	54	37	17	84	9	9	80	21	109	6	136	558
	**55.9%**	17.7%	15.4%	11.3%	4.7%	3.2%	9.7%	6.6%	3.0%	15.1%	1.6%	1.6%	14.3%	3.8%	19.5%	1.1%	24.4%	
Acute gastrointestinal diseases	115	**205**	63	30	40	18	39	26	15	73	4	7	66	16	68	6	95	417
	27.6%	**49.2%**	15.1%	7.2%	9.6%	4.3%	9.4%	6.2%	3.6%	17.5%	1.0%	1.7%	15.8%	3.8%	16.3%	1.4%	22.8%	
Allergies	142	76	**387**	74	59	24	103	67	13	149	11	12	130	30	169	7	352	1032
	13.8%	7.4%	**37.5%**	7.2%	5.7%	2.3%	10.0%	6.50%	1.30%	14.4%	1.10%	1.20%	12.6%	2.90%	16.4%	0.70%	34.10%	
Chronic resp. diseases	74	40	83	**187**	32	22	44	50	4	60	7	14	84	26	109	7	207	548
	13.5%	7.3%	15.1%	**34.1%**	5.8%	4.0%	8.0%	9.1%	0.7%	10.9%	1.3%	2.6%	15.3%	4.70%	19.9%	1.30%	37.8%	
Chronic gastrointestinal diseases	30	42	42	25	**144**	11	25	19	2	37	4	3	55	9	67	3	75	280
	10.7%	15.0%	15.0%	8.9%	**51.4%**	3.9%	8.9%	6.8%	0.7%	13.2%	1.4%	1.1%	19.6%	3.2%	23.9%	1.1%	26.8%	
Diabetes mellitus	34	28	34	26	13	**98**	20	47	7	24	8	8	49	12	89	6	204	425
	8.0%	6.6%	8.0%	6.1%	3.1%	**23.1%**	4.7%	11.1%	1.6%	5.6%	1.9%	1.9%	11.5%	2.8%	20.9%	1.40%	48.0%	
Skin diseases	73	48	81	33	34	13	**223**	37	8	83	6	13	71	11	98	6	164	521
	14.0%	9.2%	15.5%	6.3%	6.5%	2.5%	**42.8%**	7.1%	1.5%	15.9%	1.2%	2.5%	13.6%	2.1%	18.8%	1.2%	31.5%	
Cardiovascular diseases	89	55	96	61	49	55	63	**196**	9	82	17	14	117	32	202	5	423	936
	9.5%	5.9%	10.0%	6.5%	5.2%	5.9%	6.7%	**20.9%**	1.0%	8.8%	1.8%	1.5%	12.5%	3.4%	21.6%	0.5%	45.2%	
Children’s diseases	52	39	19	8	10	9	18	14	**29**	26	3	2	31	2	32	2	36	139
	37.4%	28.1%	13.7%	5.8%	7.2%	6.5%	12.9%	10.1%	**20.9%**	18.7%	2.2%	1.4%	22.3%	1.4%	23.0%	1.4%	25.9%	
Headache disorders	112	75	113	44	45	19	78	38	16	**374**	10	16	123	28	167	4	220	761
	14.7%	9.9%	14.8%	5.8%	5.9%	2.5%	10.2%	5.0%	2.1%	**49.1%**	1.3%	2.1%	16.2%	3.7%	21.9%	0.5%	28.9%	
Cancer	20	12	8	16	7	5	11	15	1	14	**39**	5	16	6	46	3	73	165
	12.1%	7.3%	4.8%	9.7%	4.2%	3.0%	6.7%	9.1%	0.6%	8.5%	**23.6%**	3.0%	9.7%	3.6%	27.9%	1.8%	44.2%	
Neurological diseases	12	14	21	18	13	9	20	15	3	27	6	**45**	24	11	50	3	59	172
	7.0%	8.1%	12.2%	10.5%	7.6%	5.2%	11.6%	8.7%	1.7%	15.7%	3.5%	**26.2%**	14.0%	6.4%	29.1%	1.7%	34.3%	
Mental disorders	98	76	85	64	58	31	71	55	8	124	10	10	**361**	31	172	10	274	820
	12.0%	9.3%	10.4%	7.8%	7.1%	3.8%	8.7%	6.7%	1.0%	15.1%	1.2%	1.2%	**44.0%**	3.80%	21.0%	1.20%	33.4%	
Thyroid diseases	67	47	75	42	22	18	47	41	5	88	8	19	92	**95**	138	10	231	587
	11.4%	8.0%	12.8%	7.2%	3.7%	3.1%	8.0%	7.0%	0.9%	15.0%	1.4%	3.2%	15.7%	**16.2%**	23.5%	1.7%	39.4%	
Musculoskeletal pain	128	77	115	82	71	43	99	84	12	153	20	27	163	45	**531**	10	353	1085
	11.8%	7.1%	10.6%	7.6%	6.5%	4.0%	9.1%	7.7%	1.1%	14.1%	1.8%	2.5%	15.0%	4.1%	**48.9%**	0.9%	32.5%	
Others	11	6	12	7	7	8	7	10	2	9	1	1	19	3	21	**42**	39	118
	9.3%	5.1%	10.2%	5.9%	5.9%	6.8%	5.9%	8.5%	1.7%	7.6%	0.8%	0.8%	16.1%	2.5%	17.8%	**35.6%**	33.1%	
Count	312	205	387	187	144	98	223	196	29	374	39	45	361	95	531	42	1279	3067

Cross-tabulation of reported conditions (rows) against conditions for which TCIM was used (columns). Percentages based on total cases per row. Bold values indicate TCIM use for corresponding condition (diagonal cells), not statistical significance (e.g., 55.9% [312/558] with acute respiratory illness). The total of 3,067 refers to respondents reporting at least one illness; multiple responses were permitted

**Fig. 2:**
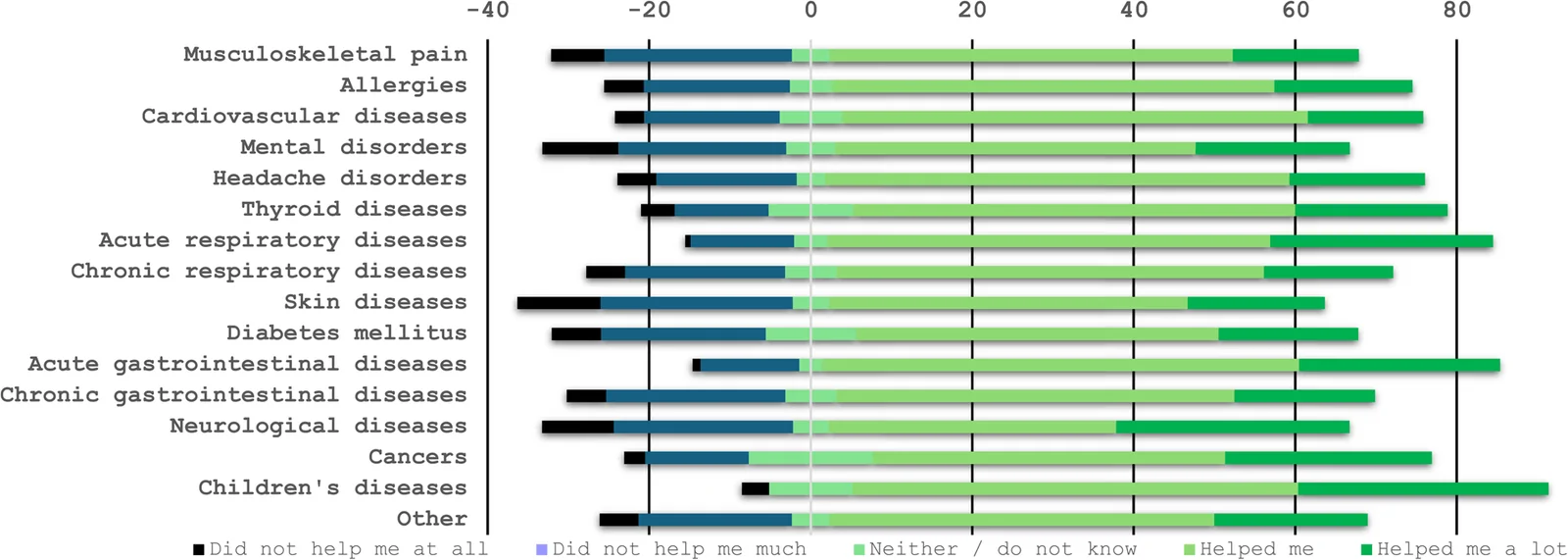
To what extent has TCIM helped you with the following illnesses?

A mean of 70.8% (range 61.4–86.2%) of TCIM users reported benefit from TCIM treatment. This was highest for childhood illness (86.2%), acute gastrointestinal illness (83.9%) and acute respiratory illness (82.4%); lowest –but still above 60%– for skin conditions (61.4%), neurological illness (64.5%) and musculoskeletal pain (65.5%) (Fig. 2).

Acute respiratory and acute gastrointestinal illnesses were the only illnesses in the top three illnesses for both TCIM use and TCIM benefit.

**Fig. 3:**
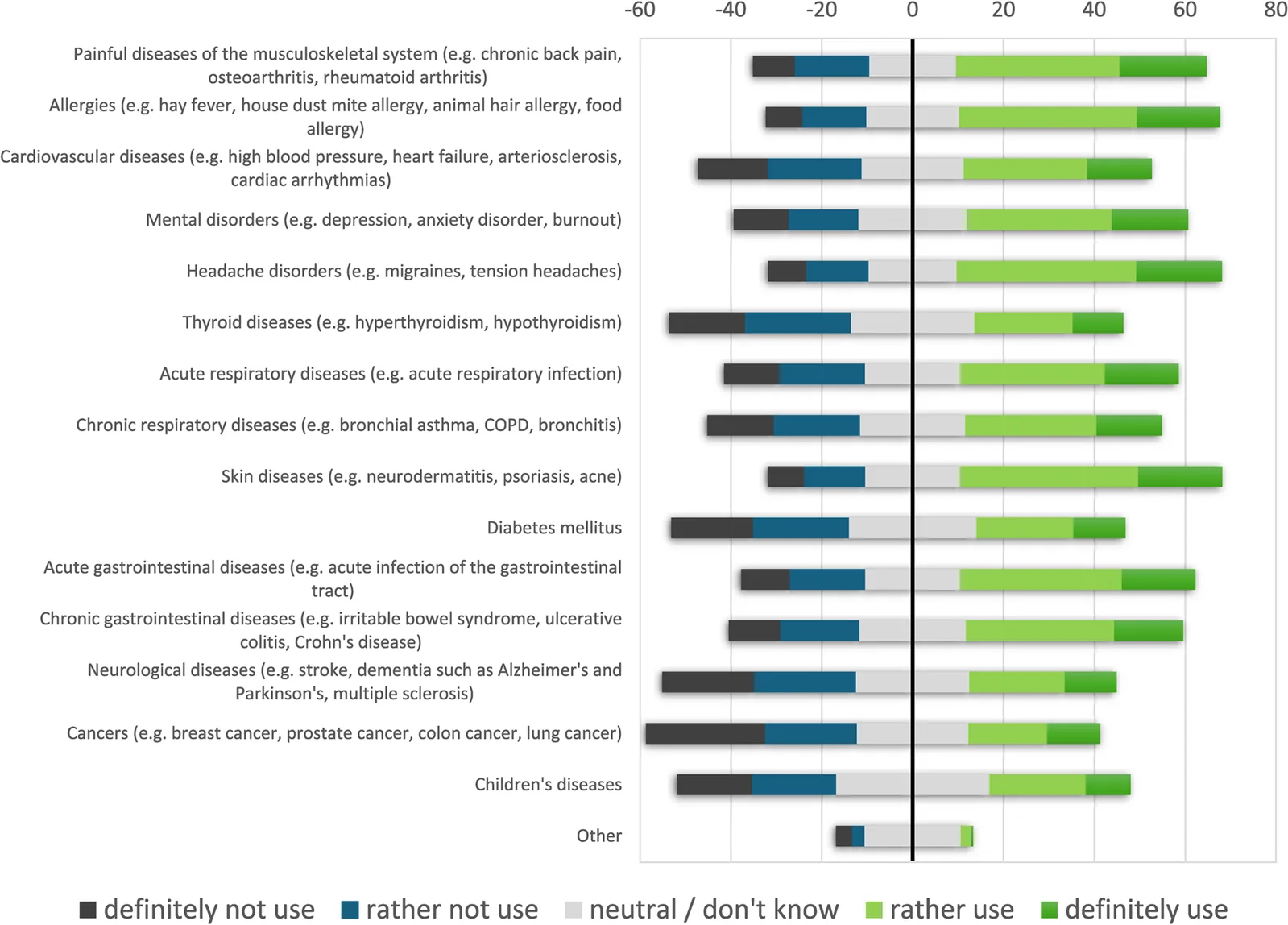
To what extent would you use TCIM for the following illnesses?

When asked if respondents would use TCIM in the future, a mean of 41.9% of all respondents would definitely or rather use TCIM (Fig. 3). The highest anticipated TCIM use was for headaches (58.4%), allergies (57.5%), skin conditions (57.7%), musculoskeletal pain (55.2%) and acute gastrointestinal illnesses (51.8%). Anticipated TCIM use was lowest for cancer (29.0%), thyroid illness (32.8%), neurological illness (32.4%) and diabetes (32.8%).

Headaches, acute gastrointestinal illness and musculoskeletal pain were in the top five illnesses for both actual TCIM use and anticipated TCIM use.

For HRQoL, a mean index value of 0.97 was achieved in individuals with excellent HRQoL, 0.95 in individuals with very good, 0.89 with good, 0.67 with fair, and 0.35 with poor HRQoL.

**Fig. 4:**
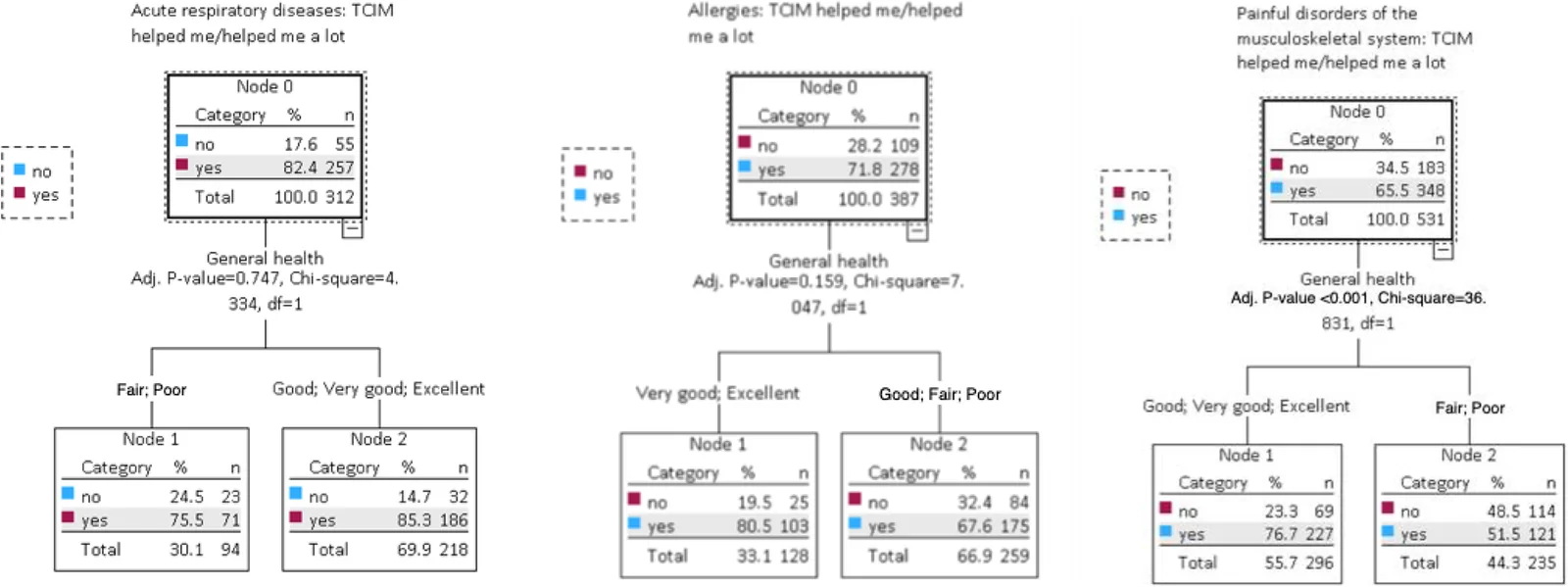
Self-experienced benefit from TCIM depending on health-related quality of life (general health status)

In the sub-analyses for acute respiratory illness, allergy and musculoskeletal pain, respondents of different general health status benefited from TCIM. However, a significantly lower benefit was experienced by individuals with musculoskeletal pain in poor or fair general health than those in better health but there was no significant difference for those with acute respiratory illness or with allergy (Fig. 4).

**Fig. 5:**
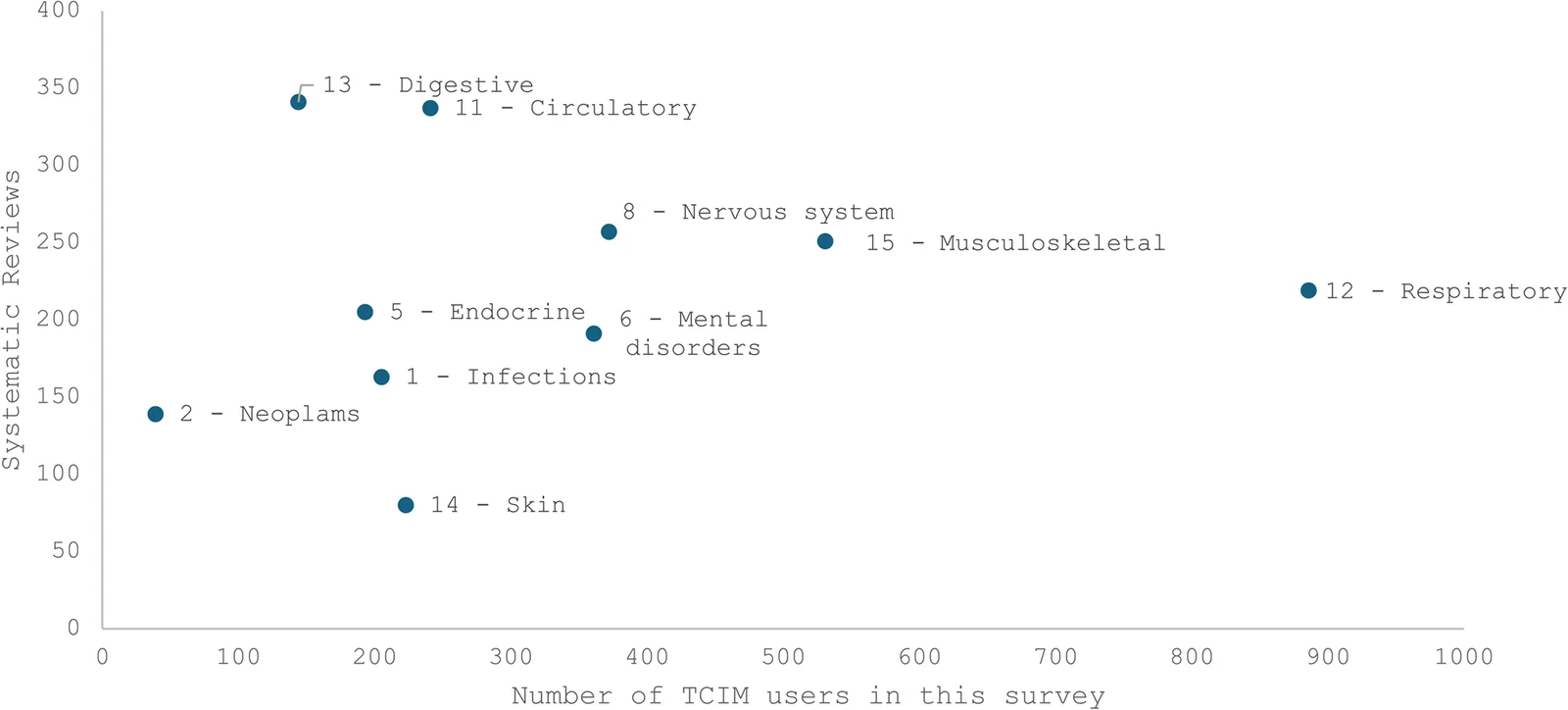
TCIM users and TCIM research activity. Legend: ICD-11 categories. Only categories included in survey questionnaire are displayed

No correlation could be observed between the number of TCIM users for a given ICD-11 disease category and the global research activity in the corresponding disease category (see scatter plot, Fig. 5).

## Discussion

TCIM was frequently used for both acute and chronic conditions, with considerable variations across diseases. This finding is notable because TCIM is often considered particularly relevant for chronic diseases [10]. When considering both highest relative use and highest experienced benefit, acute respiratory and acute gastrointestinal illnesses ranked at the top, further highlighting the relevance of TCIM for acute relief.

TCIM use for cancer (23.6%) in our sample of 165 cancer cases was surprisingly low. It is generally thought that people with cancer have high demand for TCIM—51% in one systematic review, even higher for breast cancer [11]—triggering significant attention in integrative oncology care and research [12]. In any case, the strong emphasis on integrative oncology within the European TCIM landscape, especially at universities, does not seem to align with the areas where TCIM is most widely used by the population.

TCIM use was also low in pediatric illnesses (20.9%) but our sample of 139 reports of childhood illness is implausibly low – all children face illness at some point – likely due to recall bias or a lack of importance given by respondents to long past childhood illnesses. Other surveys have reported high TCIM use in children [13]. 

The sub-analysis of individuals with three specific conditions—acute respiratory illness, allergies, and musculoskeletal pain—indicates that TCIM was perceived as beneficial not only by those with mild symptoms but also those in poorer health. High self-perceived TCIM benefit was observed across varying health statuses, though those with musculoskeletal pain and lower health status benefited significantly less.

Our analysis suggests that there is no correlation between TCIM use for certain diseases and the TCIM research activity in these diseases. This is unexpected, as research priorities might be assumed to reflect population usage pattern. Of course, researchers might also consider other factors when determining their research focus such as promising research leads, favorable research environment, funding and personal interest. Another possible, though less likely explanation is that there is already sufficient evidence for what people use more commonly and experience as most beneficial. Regardless of the research distribution across diseases, it should not be forgotten that overall TCIM research activity is far from commensurate with the wide-spread TCIM use [14]. 

There may also be differences between governments’ perception of TCIM use and its actual usage. Reflecting government perspective, WHO’s 2024 global report on TCIM, governments reported the main conditions for which people use TCIM as noncommunicable diseases (76%), disease prevention (68%), palliative care (57%), rehabilitation (56%), and health promotion (55%) [2, 6]. It remains unclear to what extent governments responded to the WHO questionnaire on actual data versus perception. These discrepancies underscore the need for more robust global survey data. In any case, this global perspective on TCIM usage poorly aligns with our German survey data.

WHO Director General Dr Tedros Ghebreyesus has still given another, more policy oriented perspective that again does not align with TCIM usage observed in Germany: “Traditional, complementary and integrative medicine is especially important for preventing and treating non-communicable diseases and mental health, and for healthy aging.” [15] His perspective, however, is well aligned with the priorities of the WHO Western Pacific region which seem to have been set by considering a combination of disease burden, need for prevention, TCIM acceptance and effectiveness [10]. Policy priorities for Europe would still have to be formulated but based on our findings, a broad TCIM offering across both acute and chronic diseases rather than a highly focused offer for a few diseases, appears the most appropriate approach.

The above examples suggest that neither research nor policy priorities for disease-specific TCIM are currently aligned with its actual usage. To address this problem, TCIM usage should be given more careful consideration in the choice of research focus and when formulating policy priorities.

A comparison of our survey with others must take into consideration that TCIM surveys often differ in methodology, including sample collection, time frame of TCIM use, varying definitions of TCIM etc. The study by Kemppainen et al. resembles our survey at least in terms of geographic region and disease focus [16]. In this study, Complementary and Alternative Medicine (CAM) use was based on the 2014 European Social Survey in 21 European countries. CAM use in the last 12 months was highest for skin conditions (38.1%), back or neck pain (38.0%), allergies (36.7%), stomach or digestive system-related problems (35.7%), upper extremity pain (34.7%) and severe headaches (34.1%). For other health conditions, including cancer and depression, usage was around 30%; for diabetes only 23.6%. Overall, this study thus identified similar levels of TCIM/CAM use to ours, though with less pronounced differences across various health conditions. The authors updated their analysis based on the 2023 European Social Survey but without disease specific usage data [4]. 

A strength of our study is that it draws from an online-representative sample of the adult German population. As TCIM covers a very wide spectrum of possible treatments and approaches, a further strength is that respondents could identify TCIM through different terms.

Our study has several limitations. It is subject to the inherent limitations of online surveys, including self-selection bias (such as those with an interest in TCIM) and potential underrepresentation of populations with limited digital access or literacy.

Another limitation of our study questionnaire was that it asked about both current and past illnesses, therefore not allowing determination of self-reported prevalence nor incidence; nor was it possible to triangulate self-reported illnesses with actual health records. However, it is likely that respondents primarily reported on current or recent illnesses, as illustrated by the fact that only 139 reported childhood illnesses, despite the likelihood that all respondents experienced these at some point in their life. Reassuringly, illness rates identified by this survey were somewhat similar, though not directly comparable, to the prevalence for selected diseases identified by the German Health Update (GEDA), a population-representative health survey of the adult population in Germany, conducted by the governmental Robert Koch Institute [17]. To illustrate this, GEDA versus our survey data identified (in order of frequency) allergy (except asthma) 30.9% versus 25.4%; osteoarthritis 17.1% versus musculoskeletal pain 26.7%; depressive symptoms in last two weeks 8.3% versus mental illness 20.2%; diabetes 8.9% versus 10.5%; coronary heart disease/cardiovascular illness 5.8% versus 23%; and chronic obstructive pulmonary disease/chronic respiratory disease 6.1% versus 13.5%.

A further limitation lies in comparing German TCIM use data with a global mapping of systematic reviews. We used the recent mapping of systematic reviews of traditional medicine modalities across health conditions by Ang et al. which included 2719 systematic reviews from the period 2018–2022 (only 10 came from Germany – personal correspondence with the author) [8]. Several traditional European modalities including homeopathy and anthroposophic medicine were not included (though aromatherapy and any kind of herbal medicine were), biasing this mapping somewhat towards Asian traditional medicines. The uniqueness and rigor of this global mapping nevertheless makes it a reference point for TCIM research activity.

## Conclusions

TCIM is widely used and perceived as beneficial across both acute and chronic illnesses, including among individuals with poorer general health. Overall TCIM research activity remains disproportionate to its widespread use. Moreover, current research priorities do not align with the diseases for which TCIM is most used. Consistent with WHO’s objectives for TCIM health system integration, the German health system should respond to the high population use of TCIM by investing in rigorous research on its effectiveness, efficacy, safety and cost-effectiveness, and by ensuring access to evidence-informed TCIM services, including statutory health insurance coverage across disease areas.

## Supplementary Information

Supplementary Material 1. Table - Reclassification into ICD-11 categories.

Supplementary Material 2. Questionnaire – English translation.

## Data Availability

The raw data supporting the conclusions of this article are available on request, without undue reservation.

## Abbreviations

GEDA: German Health Update
HRQoL: Health-related quality of life
ICD-11: International Classification of Diseases, Eleventh Revision
TCIM: Traditional, complementary and integrative medicine
WHO: World Health Organization
