# Supplementary material for: Treatment choices: insights into disease-specific use and self-perceived benefits of traditional, complementary and integrative medicine in Germany
Source: BMC Complement Med Ther. 2026 Sep 29;26:282. doi: 10.1186/s12906-026-05496-y (PMC13628838; doi:10.1186/s12906-026-05496-y)
Supplement: Supplementary file 1 [file 12906_2026_5496_MOESM2_ESM.pdf]

# Supplementary Material 1: Questionnaire

# Use and Acceptance of "Naturheilkunde" and Complementary Medicine in Germany – an Online Representative Cross-sectional Study

Note: This is an English translation of the questionnaire that was used in German language in the study. (translation revised 14 February 2025 for maximum proximity to German terminology)

## A. Introduction

Dear Sir or Madam,

the research project presented in detail below examines the use and acceptance of "Naturheilkunde" and Complementary Medicine in Germany. The Charité University Outpatient Clinic for Complementary and Integrative Medicine at the Immanuel Hospital Berlin and the Institute of Social Medicine, Epidemiology and Health Economics of Charité - Universitätsmedizin Berlin is conducting a scientific survey study on this topic in cooperation with the Otto-Friedrich University of Bamberg. Please take enough time to read this information carefully. Then decide whether you would like to take part in the survey study or not.

**Declaration of consent and study information**

[Link: Study information]

O I agree to participate in this study and confirm that I have been informed in writing of all the points listed above. I have been informed in the study information about the nature, significance, scope and risks of the scientific investigation as part of the above-mentioned study.
I am aware that I can withdraw my consent to participate in this survey study at any time without giving reasons and that this will have no negative consequences for me.

[*Declaration of consent is mandatory, otherwise end of interview*]

## B. Screening

1. Are you female, male or diverse? *[Int. quota criterion]*

| | |
|---|---|
| O | Female |
| O | Male |
| O | Diverse |

2. How old are you? *[Int. quota criterion;* numerical query]

____ years old

3. What is your highest school-leaving qualification or vocational training qualification? [*Int. quota criterion*]

| | |
|---|---|
| O | No general school-leaving certificate (yet), still a pupil at a general school |
| O | Secondary (elementary, basic) school leaving certificate without completed apprenticeship/vocational training |
| O | Secondary school leaving certificate with completed apprenticeship/vocational training |
| O | Secondary school without A-levels (German: Realschulabschluss/Mittlere Reife/Oberschule) or equivalent qualification |
| O | A-levels, (technical) university entrance qualification without studies |
| O | Studies (university, college, university of applied sciences, polytechnic) |
| O | No general school-leaving certificate (yet), still a pupil at a general school |

*4.* How many people live in your household in total, including yourself? *[Int. numeric query]*

___ People

5. How many people in your household are under the age of 14? *[Int. numeric query]*

___ People

*6.* If you take all income together: "What is the total monthly net income of all household members (after deduction of taxes or social insurance)?" *[Int. single answer]*

| | |
|---|---|
| O | less than 500 Euro |
| O | 500 to under 750 euros |
| O | 750 to under 1,000 euros |
| O | 1,000 to under 1,250 euros |
| O | 1,250 to under 1,500 euros |

| | |
|---|---|
| O | 1,500 to under 1,750 euros |
| O | 1,750 to under 2,000 euros |
| O | 2,000 to under 2,250 euros |
| O | 2,250 to under 2,500 euros |
| O | 2,500 to under 3,000 euros |
| O | 3,000 to under 3,500 euros |
| O | 3,500 to under 4,000 euros |
| O | 4,000 to under 4,500 euros |
| O | 4,500 to under 5,000 euros |
| O | 5,000 euros to under 6,000 euros |
| O | 6,000 euros to under 7,000 euros |
| O | 7,000 euros to under 8,000 euros |
| O | 8,000 euros and more |

7. What is your <u>own monthly net income</u> (after deduction of taxes or social insurance)?
   *[Int. quota criterion, soft quota]*

| | |
|---|---|
| O | No own income |
| O | up to less than 500 Euro |
| O | 500 to under 750 euros |
| O | 750 to under 1,000 euros |
| O | 1,000 to under 1,250 euros |
| O | 1,250 to under 1,500 euros |
| O | 1,500 to under 1,750 euros |
| O | 1,750 to under 2,000 euros |
| O | 2,000 to under 2,250 euros |
| O | 2,250 to under 2,500 euros |
| O | 2,500 to under 3,000 euros |
| O | 3,000 to under 3,500 euros |
| O | 3,500 to under 4,000 euros |
| O | 4,000 to under 4,500 euros |
| O | 4,500 to under 5,000 euros |
| O | 5,000 euros to under 6,000 euros |
| O | 6,000 euros to under 7,000 euros |
| O | 7,000 euros to under 8,000 euros |
| O | 8,000 euros and more |

8. In which federal state do you live?
   *[Int. quota criterion]*

| | |
|---|---|
| O | Baden-Württemberg |
| O | Bavaria |
| O | Berlin |
| O | Brandenburg |
| O | Bremen |
| O | Hamburg |

| | |
|---|---|
| O | Hesse |
| O | Mecklenburg-Western Pomerania |
| O | Lower Saxony |
| O | North Rhine-Westphalia |
| O | Rhineland-Palatinate |
| O | Saarland |
| O | Saxony |
| O | Saxony-Anhalt |
| O | Schleswig-Holstein |
| O | Thuringia |

9. How large is the place where you currently live?
   [*Int. quota criterion*]

| | |
|---|---|
| O | Under 2,000 inhabitants |
| O | 2,000 to under 5,000 inhabitants |
| O | 5,000 to under 20,000 inhabitants |
| O | 20,000 to under 50,000 inhabitants |
| O | 50,000 to under 100,000 inhabitants |
| O | 100,000 to under 500,000 inhabitants |
| O | 500,000 inhabitants and more |

## C. Knowledge

10. Which source of knowledge is most important to you for medical issues?
*[Int. single answer]*

| | |
|---|---|
| O | Active Internet search (e.g. Google) |
| O | Social internet media (e.g. Instagram, Facebook, TikTok, messenger services) |
| O | Television and radio |
| O | Magazines and newspapers |
| O | Medical professionals (e.g. physicians or alternative practitioners) |
| O | Family |
| O | Friends or acquaintances |
| O | Other, namely [free text] |

11. To what extent are you familiar with the following **medical terms?** [*Int. single answer per line]*

| | I am very familiar with it | I am familiar with it | I have heard this before | I do not know |
|---|---|---|---|---|
| "Naturheilkunde" / natural therapies | O | O | O | O |
| Herbal medicines | O | O | O | O |
| Complementary Medicine | O | O | O | O |
| Integrative Medicine | O | O | O | O |
| Alternative Medicine | O | O | O | O |

12. To what extent are you familiar with these **medical procedures?** *[Int. single answer per line, randomize]*

| | Am very familiar | Am familiar | I've heard that before | I do not know |
|---|---|---|---|---|
| Acupuncture | O | O | O | O |
| Anthroposophic medicine | O | O | O | O |
| Ayurveda | O | O | O | O |
| Fasting | O | O | O | O |
| Hijama | O | O | O | O |
| Homeopathy | O | O | O | O |
| Hydrotherapy/water treatments/ Kneipp baths | O | O | O | O |
| Phytotherapy/herbal medicine | O | O | O | O |
| Manual medicine/osteopathy/chiropractic care | O | O | O | O |
| Traditional African Medicine | O | O | O | O |
| Traditional Chinese Medicine | O | O | O | O |
| Wholefood plant-based diet | O | O | O | O |

| | | | | |
|---|---|---|---|---|
| Forest bathing/forest therapy | O | O | O | O |
| Yoga | O | O | O | O |
| Stress management, relaxation techniques, meditation (Mind-Body Medicine) | O | O | O | O |
| Movement and dance therapy | O | O | O | O |
| Art and music therapy | O | O | O | O |
| Other: [Free text] | O | O | O | O |

13. Have you completed medical training or attended medical courses in the broader sense? [*Multiple answers possible but not yes and no answers at the same time]*

| | |
|---|---|
| O | Yes, nursing training / nursing studies |
| O | Yes, naturopath (German: Heilpraktiker) examination |
| O | Yes, a medical degree |
| O | Yes, a degree in pharmacy |
| O | Yes, physiotherapy training |
| O | Yes, occupational therapy training |
| O | No |
| O | No, but I have acquired some basic medical knowledge myself |
| O | Other: [Free text] |

## D. Attitudes towards “Naturheilkunde” and Complementary Medicine

14. The following is about your opinion on certain medical procedures and their applications. Please first read through the explanation of the terms and assess whether you have a positive or negative attitude towards them.

a) How is your general attitude towards **“Naturheilkunde”/natural therapies**?
Explanation: health promotion or treatments with natural healing methods, e.g. with phytotherapy, fasting and a healthy diet, exercise and a healthy lifestyle or Kneipp water treatments (hydrotherapy). *[Int. single answer]*

| 1<br>Very positive | 2<br>Mainly positive | 3<br>Neutral | 4<br>Mainly negative | 5<br>Very negative | I do not know |
|---|---|---|---|---|---|
| O | O | O | O | O | O |

b) How is your general attitude towards **Conventional Medicine?**
Explanation: the socially established "conventional medicine" taught at medical faculties. *[Int. single answer]*

| 1<br>Very positive | 2<br>Mainly positive | 3<br>Neutral | 4<br>Mainly negative | 5<br>Very negative | I do not know |
|---|---|---|---|---|---|
| O | O | O | O | O | O |

c) How is your general attitude towards **Complementary Medicine?**
Explanation: traditional diagnostic and therapeutic methods from Western culture that complement conventional medicine, but also, for example, from traditional Chinese or Indian medicine. *[Int. single answer]*

| 1<br>Very positive | 2<br>Mainly positive | 3<br>Neutral | 4<br>Mainly negative | 5<br>Very negative | I do not know |
|---|---|---|---|---|---|
| O | O | O | O | O | O |

d) How is your general attitude towards **Integrative Medicine?**
Explanation: combination of conventional medicine with evidence based “Naturheilkunde” and Complementary Medicine. *[Int. single answer]*

| 1<br>Very positive | 2<br>Mainly positive | 3<br>Neutral | 4<br>Mainly negative | 5<br>Very negative | I do not know |
|---|---|---|---|---|---|
| O | O | O | O | O | O |

e) How is your general attitude towards **<u>Alternative Medicine?</u>**
Explanation: non-scientifically supported healing methods which, by definition, are used as an alternative to conventional medicine, often because conventional methods are rejected.
*[Int. single answer]*

| 1 Very positive | 2 Mainly positive | 3 Neutral | 4 Mainly negative | 5 Very negative | I do not know |
|---|---|---|---|---|---|
| O | O | O | O | O | O |

*15.* How important is "Naturheilkunde" for your health?
*[Int. single answer]*

| 1 Very important | 2 Somewhat important | 3 Neutral | 4 Somewhat unimportant | 5 Completely unimportant | I do not know |
|---|---|---|---|---|---|
| O | O | O | O | O | O |

## E. Application

16. How often do you currently use “Naturheilkunde”/natural therapies/complementary medicine/integrative medicine or alternative medicine?
*[Int. single answer]*

| | |
|---|---|
| O | Daily |
| O | Several times a week |
| O | Several times a month |
| O | Several times a year |
| O | Less frequently |
| O | Never |

17. To what extent do the following statements apply to you?
*[Int. single answer per line]*

| | Yes | No | Don't know |
|---|---|---|---|
| I have been using interventions of “Naturheilkunde”/natural therapies/complementary medicine/integrative medicine or alternative medicine in the last 12 months. | O | O | O |
| I am currently using interventions of “Naturheilkunde”/natural therapies/complementary medicine/integrative medicine or alternative medicine. | O | O | O |
| Other members of my household are currently using interventions of “Naturheilkunde”/natural therapies/complementary medicine/integrative medicine or alternative medicine. | O | O | O |
| I intend to use interventions of “Naturheilkunde”/natural therapies/complementary medicine/integrative medicine or alternative medicine. | O | O | O |

18. Do you currently suffer from the following illnesses or have you suffered from one or more of the following illnesses in the past?
Multiple answers are possible

| | |
|---|---|
| O | Acute respiratory diseases (e.g. acute respiratory tract infection) |
| O | Acute gastrointestinal diseases (e.g. acute infection of the gastrointestinal tract) |
| O | Allergies (e.g. hay fever, house dust mite allergy, animal hair allergy) |
| O | Chronic respiratory diseases (e.g. asthma, COPD) |
| O | Chronic gastrointestinal diseases (e.g. irritable bowel syndrome, ulcerative colitis, Crohn's disease) |
| O | Diabetes mellitus |
| O | Skin diseases (e.g. neurodermatitis, psoriasis, acne) |
| O | Cardiovascular diseases (e.g. high blood pressure, heart insufficiency, arteriosclerosis, arrhythmia) |
| O | Pediatric diseases |

| | |
|---|---|
| O | Headache diseases (e.g. migraine, tension headache) |
| O | Cancer (e.g. breast cancer, prostate cancer, lung cancer) |
| O | Neurological diseases (e.g. stroke, dementia such as Alzheimer's and Parkinson's, multiple sclerosis) |
| O | Psychological diseases (e.g. depression, anxiety) |
| O | Thyroid diseases (e.g. hyperthyroidism, hypothyroidism) |
| O | Pain diseases of the musculoskeletal system (e.g. chronic back pain, osteoarthritis, slipped disc) |
| O | Other: [Free text] |
| O | None of the above |

19. To what extent **would** you **use** "Naturheilkunde"/natural therapies/complementary medicine/integrative medicine or alternative medicine for the following illnesses?
*[Int. single answer per line Mandatory answer per line except item "Other" no mandatory answer; ]*

| | | Definitely use | rather use | Rather not use | Do not use under any circumstances | Neutral /do not know |
|---|---|---|---|---|---|---|
| 1 | Acute respiratory diseases (e.g. acute respiratory tract infection) | O | O | O | O | O |
| 2 | Acute gastrointestinal diseases (e.g. acute infection of the gastrointestinal tract) | O | O | O | O | O |
| 3 | Allergies (e.g. hay fever, house dust mite allergy, animal hair allergy) | O | O | O | O | O |
| 4 | Chronic respiratory diseases (e.g. asthma, COPD) | O | O | O | O | O |
| 5 | Chronic gastrointestinal diseases (e.g. irritable bowel syndrome, ulcerative colitis, Crohn's disease) | O | O | O | O | O |
| 6 | Diabetes mellitus | O | O | O | O | O |
| 7 | Skin diseases (e.g. neurodermatitis, psoriasis, acne) | O | O | O | O | O |
| 8 | Cardiovascular diseases (e.g. high blood pressure, heart insufficiency, arteriosclerosis, arrhythmia) | O | O | O | O | O |
| 9 | Pediatric diseases | O | O | O | O | O |
| 10 | Headache diseases (e.g. migraine, tension headache) | O | O | O | O | O |
| 11 | Cancer (e.g. breast cancer, prostate cancer, colon cancer, lung cancer) | O | O | O | O | O |

| | | | | | | |
|---|---|---|---|---|---|---|
| 12 | Neurological diseases (e.g. stroke, dementia such as Alzheimer's and Parkinson's, multiple sclerosis) | O | O | O | O | O |
| 13 | Psychological diseases (e.g. depression, anxiety) | O | O | O | O | O |
| 14 | Thyroid diseases (e.g. hyperthyroidism, hypothyroidism) | O | O | O | O | O |
| 15 | Pain diseases of the musculoskeletal system (e.g. chronic back pain, osteoarthritis, slipped disc) | O | O | O | O | O |
| 16 | Other: [Free text] | | | | | |

20. For which illnesses have you already used "Naturheilkunde"/natural therapies/complementary medicine/integrative medicine or alternative medicine? Multiple answers possible

| | |
|---|---|
| O | *[Int. list of diseases mentioned by respondent in Item 18]* |
| O | None of the methods mentioned used |

21. To what extent has "Naturheilkunde"/natural therapies/complementary medicine/integrative medicine or alternative medicine helped you with the following illnesses? *[Int. single answer per line]*

| | Helped me a lot | Helped me | Did not help me much | Did not help me at all | Neither / do not know |
|---|---|---|---|---|---|
| [List of diseases mentioned by respondent in F20] | | | | | |

22. In your opinion, who has the greatest expertise in the field of has "Naturheilkunde"/natural therapies/complementary medicine/integrative medicine or alternative medicine? *[Int. single answer]*

| | |
|---|---|
| O | Medical Doctor |
| O | Alternative practitioner (German: Heilpraktiker) |
| O | Pharmacist |
| O | Patient |
| O | Other: [Free text] |
| O | No opinion |

23. To what extent do you consider “Naturheilkunde”/natural therapies/complementary medicine/integrative medicine or alternative medicine to be optimal?
*[Int. single answer]*

*I consider* “Naturheilkunde”/natural therapies/complementary medicine/integrative medicine or alternative medicine to be optimal...

| | |
|---|---|
| O | ...alone, without conventional medicine [in the sense of alternative medicine]. |
| O | ...in combination with conventional medicine [in the sense of integrative medicine]. |
| O | ...as a complement to conventional medicine [in the sense of complementary medicine]. |
| O | ...only in exceptional medical situations or individual cases. |
| O | ...no, should not be used at all. |
| O | ...undecided/no opinion. |

24. How important are the reasons listed for using “Naturheilkunde”/natural therapies/complementary medicine/integrative medicine or alternative medicine to you?
*[Int. randomize, single answer per item; only ask if one of the procedures has already been used F20]*

| 5<br>Very important | 4 | 3 | 2 | 1<br>Completely unimportant |
|---|---|---|---|---|
| O | O | O | O | O |

| | |
|---|---|
| 1 | Fewer side effects than with conventional medicine |
| 2 | To reduce the side effects of conventional medicines |
| 3 | Chances of recovery are better |
| 4 | Family, friends or acquaintances have had good experiences |
| 5 | I myself have had good experiences |
| 6 | This strengthens my health competence / self-treatment competence |
| 7 | The advice of my attending doctor |
| 8 | I have heard about it in the media |
| 9 | I am doing it out of health-related desperation |
| 10 | I am doing it out of curiosity about these procedures |
| 11 | I don't think much of conventional medicine |
| 12 | I have had bad experiences with conventional medicine |
| 13 | Don't know |
| 14 | Other [free text] |

25. How much do the aspects listed below influence your decision to choose a treatment method?
*[Int. 5-point scale, single response per item; compulsory response per line except item "Other" no compulsory response]*

| 5<br>Influences my decision enormously or extremely strongly | 4 | 3 | 2 | 1<br>Does not influence my decision at all |
|---|---|---|---|---|

| | | | | |
|---|---|---|---|---|
| O | O | O | O | O |

| | |
|---|---|
| O | Results of scientific studies |
| O | Doctor's recommendations |
| O | Personal recommendations |
| O | Experiences of family, friends and acquaintances |
| O | Own previous experience |
| O | Other [free text] |

26. Do you currently take conventional medication on a regular daily basis?
    *[Single answer]*

| | |
|---|---|
| O | Yes |
| O | No |

27. Do you currently take dietary supplements, vitamin supplements or herbal remedies on a regular daily basis?
    *[Int. single answer]*

| | |
|---|---|
| O | Yes |
| O | No |

## F. State of health

Items 28-31. The EQ-5D-5L quality of life questionnaire is here only included as a placeholder (for copyright reasons).

## G. Evaluation of medical procedures

32. How effective do you think the following medical procedures are? Please rate the following procedures.
*[Int. single answer; mandatory information per line]*

| | Very effecti ve | Mainly effectiv e | Partly effectiv e | Rather less effectiv e | Not at all effecti ve | Don't know |
|---|---|---|---|---|---|---|
| Conventional medicine | O | O | O | O | O | O |
| "Naturheilkunde"/ natural therapies | O | O | O | O | O | O |
| Complementary medicine | O | O | O | O | O | O |
| Integrative medicine | O | O | O | O | O | O |
| Alternative medicine | O | O | O | O | O | O |

33. "Naturheilkunde"/natural therapies/complementary medicine/integrative medicine or alternative medicine is often described as unscientific. Would you agree with this assessment in principle?
*[Int. single answer]*

| | |
|---|---|
| O | Yes, absolutely |
| O | Partial |
| O | Undecided |
| O | Rather no |
| O | By no means |

34. In your opinion, which of the following answers is correct?

Drugs/medicines used in conventional medicine... Multiple answers possible

| | |
|---|---|
| O | ...I consider them to be the best means of treating illnesses. |
| O | ...I use them sparingly and carefully. |
| O | ...I only use them if I can experience their benefits. |
| O | ...I view it with skepticism because it is primarily about profit. |
| O | ...I essentially reject it because I reject conventional medicine. |
| O | other |

35. In your opinion, should there be more research into “Naturheilkunde”/natural therapies and complementary medicine?

*[Int. single answer]*

| | |
|---|---|
| O | Yes |
| O | No |
| O | Don't know / No opinion |

36. In your opinion, which of the following answers is correct?

In my view, the main reason for research in the field of “Naturheilkunde”/natural therapies/ and complementary medicine is...

*[Int. single answer]*

| | |
|---|---|
| O | ... that there is more evidence of effectiveness. |
| O | ... to please politics and industry. |
| O | ... to find new healing methods. |
| O | ... to establish these methods in society. |
| O | ... - none. Research in the field of “Naturheilkunde”/Complementary Medicine is superfluous. |
| O | ... another reason, namely: [free text] |
| O | Don't know / no opinion |

37. In your opinion, which of the following answers is correct?

The costs of “Naturheilkunde” / Complementary Medicine should be covered by health insurance.

*[Int. single answer]*

| | |
|---|---|
| O | In any case |
| O | Rather yes |
| O | Draw |
| O | In individual cases |
| O | No |

## H. Assessment of Covid-19 and TCIM use

Items 38-40. These will be published separately.

## I. Nutrition

Items 41-48. These will be published separately.

## J. Ayurveda

Items 49-53. These will be published separately.

## K. Attitude and behavioral issues

Below we have some general attitude and behavioral questions

54. Would you describe yourself as spiritual?
*[Int. single answer]*

| | |
|---|---|
| O | *Yes, very* |
| O | *Yes, something* |
| O | *Neither* |
| O | *Rather not* |
| O | *Not at all* |
| O | *Don't know* |

55. Please answer the following sentence with the following answer options.
"Digital technologies play a central role in my life." *[Int. single answer]*

| | |
|---|---|
| O | Fully applies |
| O | Mainly applies |
| O | Neutral |
| O | Rather not applicable |
| O | Does not apply at all |
| O | Draw |

56. Which political party is closest to you personally?
*[Int. single answer]*

| | |
|---|---|
| O | CDU |
| O | CSU |
| O | FDP |
| O | Bündnis 90/Die Grünen |
| O | SPD |
| O | Die Linke |
| O | AfD |

| | |
|---|---|
| O | Other political parties, namely__________________ |
| O | Not specified |

57. Please answer the following sentence with the following answer options. "To what extent do you trust the democratic structures in Germany?" *[Int. single answer]*

| | |
|---|---|
| O | I trust without reservation |
| O | I do mainly trust |
| O | Neither trust nor no trust |
| O | Rather lack confidence |
| O | Have no confidence at all |

## L. Sinus milieu indicator ®

Items 58-86. These will be published separately.

## M. Sociodemographics

Finally, we have a few last questions for you:

87. Please tell me your marital status. Are you...?

*[Int. single answer]*

| | | |
|---|---|---|
| 1 | Single | O |
| 2 | Married | O |
| 3 | Divorced | O |
| 4 | Widowed | O |

88. Do you have children, regardless of whether they live with you in your household or not?

*[Int. single answer]*

| | | |
|---|---|---|
| 1 | Yes | O |
| 2 | No | O |

89. Are you employed? Which of this list applies to you?

*[Int. single answer]*

| | | |
|---|---|---|
| O | Apprentice, trainee | ➔ 62 |
| O | Pupils | ➔ 62 |
| O | Student | ➔ 62 |
| O | Fully employed | ➔ F61a |
| O | Partially employed | ➔ F61a |
| O | Currently unemployed or looking for work | ➔ F61b |
| O | On parental leave | ➔ F61b |
| O | In retraining | ➔ F61b |
| O | Pensioner, retiree | ➔ F61b |
| O | Not working | ➔ F61b |

*90.* a)[ Int. if employed]
What position do you hold in your current profession? (if employed)
*[Int. single answer]*

b)*[ Int. if not (no longer) employed]*
What position did you hold in your previous job?
*[Int. single answer]*

| | |
|---|---|
| O | Self-employed without employees |
| O | Self-employed with up to 5 employees (including yourself) |
| O | Self-employed with 6 to 49 employees (including yourself) |
| O | Self-employed with 50 or more employees (including yourself) |
| O | Freelancer without employees |
| O | Freelancer with at least 1 employee |
| O | Ordinary employee |
| O | Intermediate employee |
| O | Qualified employee |
| O | Senior employee |
| O | Civil servant(s), elementary service |
| O | Civil servant(s) intermediate service |
| O | Senior civil servant(s) |
| O | Official(s) Higher Service |
| O | Worker: Simple work (mainly physical strength) |
| O | Worker: Difficult work (mainly skill) |
| O | Worker: skilled worker, foreman, foreman, journeyman |
| O | Self-employed farmer |
| O | have never been employed |

91. What position do your parents hold in your profession or did they hold in your previous profession?
*[Int. single answer per parent (column)]*

| Father | Mother | |
|---|---|---|
| O | O | Self-employed without employees |
| O | O | Self-employed with up to 5 employees |
| O | O | Self-employed with 6 to 49 employees |
| O | O | Self-employed with 50 or more employees |
| O | O | Freelancer without employees |
| O | O | Freelancer with at least 1 employee |
| O | O | Ordinary employee |
| O | O | Intermediate employee |
| O | O | Qualified employee |
| O | O | Senior employee |
| O | O | Civil servant(s), elementary service |
| O | O | Civil servant(s) intermediate service |
| O | O | Senior civil servant(s) |
| O | O | Official(s) Higher Service |
| O | O | Worker: Simple work (mainly physical strength) |
| O | O | Worker: Difficult work (mainly skill) |
| O | O | Worker: skilled worker, foreman, foreman, journeyman |
| O | O | Self-employed farmer |
| O | O | have never been employed |

92. Which nationality(ies) do you have? (incl. dual nationality)
*[Int. multiple answers possible]*

| **Country** | O | **Country** | O |
|---|---|---|---|
| Germany | O | Netherlands | O |
| Turkey | O | Norway | O |
| Italy | O | Austria | O |
| Albania | O | Poland | O |
| Andora | O | Portugal | O |
| Australia | O | Romania | O |
| Belgium | O | Russia | O |
| Bosnia-Herzegovina | O | San Marino | O |
| Bulgaria | O | Sweden | O |
| Denmark | O | Switzerland | O |
| Estonia | O | Serbia | O |
| Finland | O | Slovakia | O |
| France | O | Slovenia | O |
| Greece | O | Spain | O |
| Great Britain | O | Czech rebuplication | O |
| Ireland | O | Ukraine | O |
| Iceland | O | Hungary | O |
| Croatia | O | Vatican | O |
| Latvia | O | Belarus | O |
| Lichtenstein | O | Cyprus | O |
| Lithuania | O | Stateless | O |
| Luxembourg | O | Other: ______________ | O |
| Malta | O | | |
| Macedonia | O | | |
| Moldova | O | | |
| Monaco | O | | |
| Montenegro | O | | |

93. Were both of your parents born in Germany?
*[Int. single answer]*

| | |
|---|---|
| O | No, just my father |
| O | No, just my mother |
| O | Yes, my father and mother are from Germany |
| O | Don't know |

94. Do you belong to a religious community?
*[Int. single answer]*

| | |
|---|---|
| Yes, Catholic | O |
| Yes, Protestant | O |
| Yes, Muslim | O |
| Yes, Buddhist | O |
| Yes, Hindu | O |
| Yes, Jewish | O |
| Yes, other religious community, namely;_____________ | |
| No, no religious affiliation/atheist | O |

Thank you for your participation!
