# Supplementary material for: Treatment choices: insights into disease-specific use and self-perceived benefits of traditional, complementary and integrative medicine in Germany
Source: BMC Complement Med Ther. 2026 Sep 29;26:282. doi: 10.1186/s12906-026-05496-y (PMC13628838; doi:10.1186/s12906-026-05496-y)
Supplement: Supplementary file 2 [file 12906_2026_5496_MOESM1_ESM.docx]

| **Survey classification** | **Reclassification into ICD-11 categories** |
| --- | --- |
| Acute respiratory diseases (e.g. acute respiratory infection) | Diseaes of the respiratory system |
| Acute gastrointestinal illnesses (e.g. acute infection of the gastrointestinal tract) | Certain infectious or parasitic diseases |
| Allergies (e.g. hay fever, house dust mite allergy, animal hair allergy, food | Diseases of the respiratory system |
| Chronic respiratory diseases (e.g. bronchial asthma, COPD, bronchitis) | Diseaes of the respiratory system |
| Chronic gastrointestinal diseases (e.g. irritable bowel syndrome, ulcerative colitis, Chron’s disease) | Diseases of the digestive system |
| Diabetes mellitus | Endocrine, nutritional, or metabolic diseases |
| Skin diseases (e.g. atopic dermatitis, psoriasis, acne) | Diseases of the skin and subcutaneous tissue |
| Cardiovascular diseases (e.g. hypertension, heart failure, arteriosclerosis) | Diseases of the circulatory system |
| Children's diseases |  |
| Headache disorders (e.g. migraines, tension headaches) | Diseases of the nervous system |
| Cancer diseases (e.g. breast cancer, prostate cancer, colon cancer, lung cancer) | Neoplasms |
| Neurological diseases (e.g. stroke, dementia such as Alzheimer's and Parkinson's) - priority given to stroke | Diseases of the circulatory system |
| Mental illnesses (e.g. depression, anxiety disorder, burnout) | Mental and behavioural disorders |
| Thyroid diseases (e.g. hyperthyroidism, hypothyroidism) | Endocrine, nutritional and metabolic diseases |
| Musculoskeletal pain (e.g. chronic back pain, rheumatoid arthritis) | Diseases of the musculoskeletal system and connective tissue |
